# Examining and Licensing Stallions for the Stud1Presented before the American Veterinary Medical Association, Detroit, Mich., Sept., 1900.

**Published:** 1900-09

**Authors:** J. I. Gibson

**Affiliations:** Denison, Iowa


### EXAMINING AND LICENSING STALLIONS FOR THE STUD.[1]

By J. I. Gibson, V.S.,
DENISON, IOWA.

This subject is of the greatest importance to the men whose time and money are invested in any line of the equine industry of this great country. The inspection of stallions must carry with it the proper selection of brood-mares to insure success and the elevation of our general standard of breeding. The production of a good horse is not an accident. Horses are, nine times out of ten, as good as they were designed to be by the breeder who selected the sire and dam. A good horse-breeder studies every point of strength or weakness in stallion and mare, taking care to have one strong where the other may be weak, and expects by such careful selection to develop just the horse he planned for, and he is seldom disappointed, because he has the correct theory of breeding.

In order to succeed in breeding, we must breed and cultivate the distinct market classes, and get rid of the idea of an all or general-purpose horse. This line of talk is not especially interesting to the average veterinarian, but let me remind you that if veterinary inspection should be instituted a great many veterinarians would awaken to the fact that they are not qualified

[1] Presented before the American Veterinary Medical Association, Detroit, Mich., Sept., 1900.

to give good advice or proper instructions to the ambitious breeders. They would realize that they had spent too much time studying the horse in disease, and had not devoted time enough to the study of the perfect horse. We, as veterinarians, should not only be students of the principles of good breeding and selection, but should insist that all veterinary colleges maintain a department in which is taught the classifying and judging of the distinct classes of types of horses demanded by the markets as well as the principles of good breeding as applied to all domestic animals. Experience has taught us that to be a successful veterinarian in practice one must be a good judge of any and all classes of horses; for the veterinarian who is not a good judge of a horse in health will have trouble in convincing the owner that he is qualified to treat the horse when sick.

Heredity has a great deal to do with successful as well as unsuccessful breeding. By heredity many good characteristics are sure to be transmitted, and the breeder should be able to foresee defects and avoid their reproduction. By inspecting stallions and carefully classifying and selecting mares, we can prevent a great many weaknesses and objectionable characteristics, and while so doing can plan to develop the desired size, color, bone, and disposition in every class of horse we raise. The man who breeds for speed only cannot be governed by fixed rules, therefore, he and his sire of speed must in a sense be excluded from this paper.

This Association should prepare a scale of points to be adopted by all the fair associations in the country, and horses should be scored for points, just as hogs are. This would force exhibitors to build or breed each horse according to the ideal scale adopted for the class or type to which such horse belongs.

In order that we may hasten the desired status in horse-breeding we should seek to bring about a rigid veterinary inspection of stallions for the stud, with the licensing of all that pass a satisfactory examination, and the emasculation by the examining veterinarians, without additional fee, of all rejected on such examination.

1. The stallion should undergo a careful examination as to health and freedom from all contagious and infectious diseases, paying particular attention to the healthy condition of the genital organs. A failure to detect such diseases would often prove a serious loss to all parties concerned. The bond of the stallion proprietor should require him to remove his stallion from the stud on the first symptom of disease of any kind, and the law should provide for the punishment of the owner of a mare which is known to be diseased to such owner, but which is brought to be bred to such licensed stallion. Such provision would afford protection to the owners of stallions, and often prevent outbreaks of contagious and infectious diseases.

2. The horse's pedigree should be looked into. The time has surely come when no grade stallions should be allowed to enter the stud. None but full bloods should be passed as eligible for breeding.

3. A careful examination as to soundness, freedom from hereditary weakness or unsoundness, and if such ailments as are transmitted by heredity be found to exist, the stallion should be condemned unless it can be clearly shown that his ancestry on both sides for two generations back were absolutely free from such hereditary weakness or unsoundness.

4. His form and proportions should be carefully taken, viz., height of body and length of legs, girth of chest and loins, form of back line, height of head when standing naturally erect, girth of cannon-bone just below the knee, and metatarsal just below the hock, weight, form of head and face as an indication of disposition; also his various gaits in action.

The examiners should be furnished with a uniform set of blanks, setting forth all the points above mentioned, and, taking a hundred for perfection in each, the examiner should give each stallion his percentage of perfection on each and every point and his general average as a whole. The examiner's fees should be paid from county or State funds, so that he would be in no way obligated to the owners of stallions. A severe penalty should be attached by law to malfeasance of any nature in office, and he should be required to give bonds for the proper conduct of his office. The owner of a stallion should be required to breed only a certain class and style of mares, such as the inspector should deem proper and define in conditions of license.

The inspector should be appointed by the Governor, and he should be an assistant to the State veterinarian; a complete record of all licenses issued for stallions, in the form of a duplicate of such licenses, should be kept in the office of the State Veterinarian, and a record of all licensed stallions in each county should be made in the office of the county recorder. The above-mentioned records would enable the State to keep a better horse-census. Each owner of licensed stallions might be required to file a report with the State Veterinarian, setting forth the number of mares bred to such licensed stallion and the number known to be pregnant on or before December 31st of each year. If any stallion be found in the stud not so licensed and recorded the law should hold the owner guilty of a misdemeanor, and he should be punished accordingly. All stallions should be examined during the month of January, and if licensed they should be re-examined for renewal of license during January of each year.

After briefly outlining the inspection, the writer recommends that the American Veterinary Medical Association pass resolutions recommending legislation in all the States and provinces requiring the licensing of stallions, subject to an examination on a scale of points as outlined above.

The writer further recommends that a committee, consisting of five members of the Association, be appointed to prepare a complete score-card for each class of stallion, and report same at next meeting.

